# Regulation of T follicular helper cells by ICOS

**DOI:** 10.18632/oncotarget.4798

**Published:** 2015-07-09

**Authors:** Andreas Hutloff

**Affiliations:** Chronic Immune Reactions, German Rheumatism Research Centre (DRFZ), a Leibniz Institute, Berlin, Germany

**Keywords:** Immunology and Microbiology Section, Immune response, Immunity

T follicular helper (TFH) cells are gatekeepers of the humoral immune response. Without help from this CD4^+^ T cell subset, B cells cannot differentiate into high-affinity memory B cells and antibody-producing long-lived plasma cells which are the basis of protective immune responses. At the same time, dysregulated TFH cell responses are causative for many autoimmune disorders (reviewed in [1]). The inducible T cell costimulator ICOS, which is structurally and functionally related to CD28, has been known for many years as an important regulator of TFH cells. ICOS knock-out mice as well as ICOS-deficient patients have only few TFH cells and very small germinal centers upon immunization, which results in severely compromised antigen-specific immunoglobulin levels and the phenotype of common variable immunodeficiency in humans (reviewed in [2]). However, the molecular mechanisms responsible for this defect were unknown until recently. Now, within the last few months a series of independent publications unraveled the complete signaling pathway of how ICOS regulates TFH cells [3–6].

**Figure 1:**
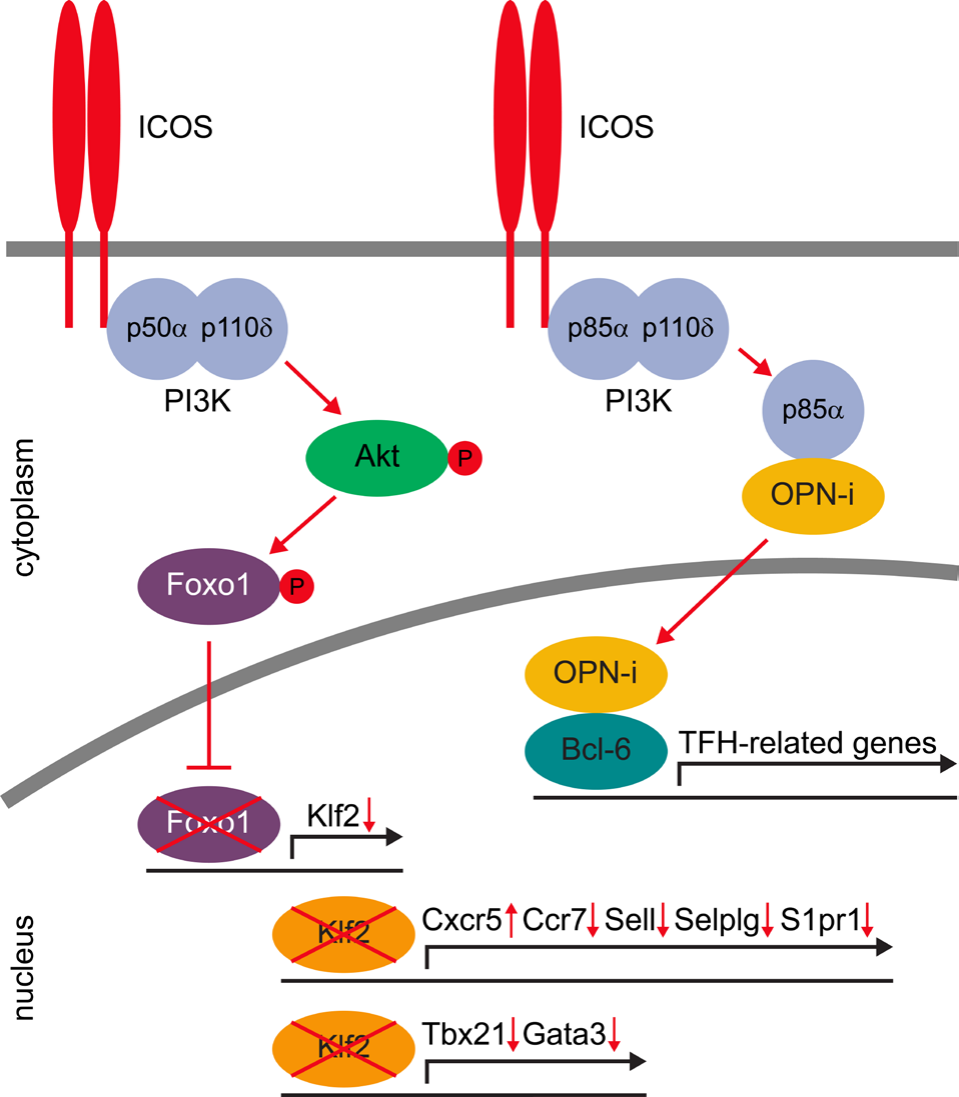
Signaling pathway for ICOS costimulation in TFH cells

ICOS exerts its costimulatory function via activation of the phosphatidylinositol-3- (PI3-) kinase which results in activation of Akt, also known as protein kinase B (Figure 1). Akt phosphorylates the transcription factor Foxo1, which is thereby retained in the cytoplasm [5, 6]. Among the many Foxo1 downstream targets, the transcription factor Klf2 turned out to be of special importance for TFH cells [4, 6]. Knock-out of Klf2 results in strongly enhanced number of TFH cells [4]. Klf2 directly binds to the promotor regions of the chemokine receptors Cxcr5 and Ccr7, the cell homing receptors Sell (encoding CD62L) and Selplg (encoding PSGL-1), and the sphingosine-1-phosphate receptor (S1pr1) [6]. Klf2 has to be expressed at low levels to maintain the typical expression pattern of homing receptors which keep TFH cells in the B cell follicle. This means that ICOS costimulation does not directly regulate any of the lineage-defining transcription factors for TFH cells like Bcl-6 and Ascl-2, but is important for the correct localization of TFH cells within secondary lymphoid organs. In addition, Klf2 promotes expression of the transcription factors T-bet and Gata3, which promote differentiation of Th1 and Th2 cells, respectively [4, 6].

Beside the ICOS - Klf2 axis, a second independent pathway exists. Although the cytoplasmic tail of ICOS preferentially recruits the p50α/p110δ isoform of PI3 kinase [2], activation of p85α is also possible. This PI3 kinase subunit can bind intracellular osteopontin (OPN-i), which facilitates nuclear translocation [3]. In the nucleus, OPN-i dimerizes with Bcl-6 and protects it from proteasomal degradation. However, this OPN-i and the above Klf2 pathway do act at different times. Upon ICOS signaling blockade, Klf2 is upregulated within a few hours and TFH cells lose their typical homing receptor pattern in less than 24 hours [6]. In contrast, complete degradation of Bcl-6 takes several days [3, 6]. Therefore, the ICOS - Klf2 axis first leads to emigration of TFH cells out of the B cell follicle (and thereby to a loss of function), whereas the osteopontin pathway acts as a second strike pathway eliminating the lineage-defining transcription factor Bcl-6.

Another important finding especially for potential therapeutic applications is that ICOS and the structurally related CD28 molecule act in different phases of TFH cell differentiation [6]. Blockade of the CD28 pathway using a soluble CTLA-4-Ig chimera (Abatacept) is already in clinical use for treatment of autoimmune disorders, whereas blockade of the ICOS pathway is under clinical evaluation. Unlike CD28, ICOS does not regulate any of the early differentiation steps of naive T cells into TFH cells, such as upregulation of Bcl-6. Instead, ICOS - but no longer CD28 - is important to maintain the phenotype of already differentiated TFH cells [6]. This unique role of ICOS for later phases of the immune reaction makes this costimulatory molecule very attractive for therapeutic intervention. For vaccination purposes, persistence of TFH cells and thereby prolongation of the germinal center reaction would be advantageous, whereas for the treatment of autoimmune diseases it is desired to remove already existing autoreactive TFH cells.

In this context, it will be important to see whether TFH cell irretrievable lose their phenotype upon ICOS costimulation blockade. This loss of TFH cell markers resembles the phenotype of TFH memory cells, which also strongly downregulate CXCR5 and Bcl-6 in the absence of antigen [7]. However, TFH memory cells are able to rapidly regain their phenotype upon secondary antigen contact. Therefore, following-up the fate of converted TFH cells upon ICOS costimulation blockade will be an important area of future research.
